# Digitalization and financial inclusion among women-led micro and small enterprises in Indonesia: an empirical perspective

**DOI:** 10.3389/fsoc.2026.1813070

**Published:** 2026-05-14

**Authors:** Diah Pranitasari, Sri Harini, Sukmo Hadi Nugroho, Susi Susilawati Harahap, Aries Sudiarso

**Affiliations:** 1 Management Department, Sekolah Tinggi Ilmu Ekonomi Indonesia Jakarta, Jakarta, Indonesia; 2 Management Department, Faculty of Economics and Business, Universitas Djuanda, Bogor, Indonesia; 3 Master of Management Program, Faculty of Economics and Business, Universitas Esa Unggul, Jakarta, Indonesia; 4 Badan Pengembangan Sumber Daya Manusia Provinsi DKI Jakarta, Jakarta, Indonesia; 5 Faculty of Defense Management, Universitas Pertahanan Indonesia, Bogor, Indonesia

**Keywords:** capacity-performance gap, digital literacy, financial inclusion, MSME performance, women’s entrepreneurship

## Abstract

**Introduction:**

This study aims to examine how digitalization and financial inclusion influence the financial performance of women-owned micro, small, and medium enterprises (MSMEs) in Indonesia, as well as to explain the social mechanisms that mediate this relationship.

**Methods:**

This study adopts a mixed-methods approach, combining quantitative analysis based on a survey of female MSME owners with qualitative insights derived from in-depth interviews. The quantitative analysis investigates the relationships among digital literacy, financial support, self-development motivation, digital marketing, and financial literacy in relation to business performance, while the qualitative analysis provides a contextual understanding of the social and institutional factors shaping women’s entrepreneurial practices.

**Results:**

The findings reveal that digitalization and financial support do not directly enhance the financial performance of women-owned MSMEs. Instead, their effects are mediated by self-development motivation, digital marketing, and financial knowledge, attitudes, and behaviors. The results also highlight the existence of a capacity–performance gap, referring to the discrepancy between improvements in individual capacity and actual business performance outcomes.

**Discussion:**

The qualitative findings indicate that structural factors, such as asset ownership, access to financial institutions, social networks, and women’s dual roles within the household, significantly affect the ability of female entrepreneurs to translate these capacities into tangible economic outcomes. This study contributes to the literature on economic sociology and women’s entrepreneurship by demonstrating that entrepreneurial success is shaped by the interaction between individual capabilities and structural conditions influencing economic opportunities, particularly in the informal economy of developing countries.

## 1 Introduction

MSMEs managed by women play a strategic role in supporting the Indonesian economy, accounting for 65% of the total number of MSMEs, which number 66 million (Badan Pusat Statistik, 2024; Maharani, 2024; Zuhdi, 2024). They absorb 97% of the workforce and contribute 61% to Gross Domestic Product (UNICEF, 2020). The number of women-owned MSMEs in Indonesia has been increasing year after year, in line with the development of the digital economy and the expansion of access to financial services. However, this increase in the number of women-owned MSMEs has not been fully matched by sustainable business capacity building (Hutabarat, 2025; Kemenpppa, 2023).

According to a report by the Central Statistics Agency, Badan Pusat Statistik (2024), although MSMEs contribute more than 60% to national GDP, the adoption rate of digital technology among women-owned MSMEs remains relatively low compared to that of men. Furthermore, data from Perrin and Hyland (2023a) shows that only around 54% of women entrepreneurs in developing countries have access to formal financial services. In Indonesia, the Ministry of Women’s Empowerment and Child Protection also reported that the digital and financial literacy gap remains a major obstacle to increasing the competitiveness of women-owned MSMEs. This empirical fact demonstrates a gap between the economic potential of women-owned MSMEs and the realization of optimal business performance.

The Indonesian government is accelerating digitalization and the national financial inclusion agenda (Anwar, 2024), However, structural and cultural challenges remain for women-owned MSMEs. Limited digital literacy, low understanding of formal financial products and services, and the burden of dual roles within the household are factors that often hinder the optimization of business performance (Arsyad and Wahira, 2025; Siahaan and Elisabeth, 2022). Sociologically, this obstacle is exacerbated by social norms that uphold the ideology of domestication, which places household responsibilities as the absolute obligation of women (Rinaldo, 2018; Setyowati, 2020). Therefore, the burden of multiple roles is not merely a technical problem of individual time management, but rather a manifestation of role conflict that is deeply rooted in social construction where digital technology often extends the professional workload into the private sphere (Kailasapathy and Metz, 2012). This reflects that digital transformation and financial inclusion have not been fully felt equally by female MSME actors (Dzulkepli, 2021; Syahnur et al., 2024).

Previous research placed digitalization and financial inclusion as variables analyzed in the form of a causal relationship model (Asmara et al., 2025; Destiari, 2024; Higón and Bonvin, 2024; Kumar et al., 2024; Murwenie et al., 2025; Octavina and Rita, 2021; Satyadewi et al., 2023; Sutopo, 2025). This approach is often deemed inadequate to fully depict the empirical dynamics experienced by women MSMEs within the local social and cultural context. A comprehensive understanding of the experiences, perceptions, and actual practices of women entrepreneurs is necessary to formulate more contextual empowerment policies and strategies.

Based on these conditions, this study seeks to analyze the asynchronous relationship between individual capacity building, digitalization, and the financial performance of women MSMEs within the context of restrictive social structures. Unlike previous research, which generally focuses on examining the relationships between variables within conceptual models, this study emphasizes empirical analysis of the digitalization and financial inclusion practices experienced by women MSMEs. This research explores how women entrepreneurs understand, access, and utilize digital technology and financial services in running their businesses.

With this approach, this research is expected to provide a more in-depth picture of the realities of women’s entrepreneurship and the structural challenges they face in promoting business sustainability. Furthermore, unlike previous research, which tends to view gender descriptively, this study integrates a sociological perspective through an analysis of role conflict and structural barriers. Thus, this research contributes to broadening the debate on digital transformation and the dynamics of work regimes in the informal economy, particularly in the context of women-owned MSMEs in Indonesia.

## 2 Literature review

### 2.1 Digital literacy and women entrepreneurship

Although it is stated that the role of women’s MSMEs in the Indonesian economy is very large, however, a number of studies show that there are still gaps in access, utilization, and the impact of digital transformation and financial inclusion felt by women MSME actors (Dzulkepli, 2021).

Low digital literacy and financial literacy can affect the ability of female MSMEs to optimally utilize digital services and financial products (Bharali and Chakravorty, 2025; Peter et al., 2025). A systematic literature review shows that women’s digital financial literacy and access to digital services are still constrained by educational disparities, sociocultural barriers, and limited access to formal financial services. These factors inhibit the full utilization of digital technology to expand market access and venture capital (Bharali and Chakravorty, 2025; Dzulkepli, 2021; Mardhiyaturrositaningsih and Hakim, 2023). The research also found that the use of digital financial facilities by women-owned MSMEs in Indonesia remains limited, with a lack of digital and financial literacy being a major barrier to the adoption of fintech applications and digital services. Social and cultural factors, such as women’s dual roles within the family, also influence the level of adoption of this technology (Muslim, 2025; Syahnur et al., 2024).

Research conducted by Peter et al. (2025), Wulan et al. (2024), and Yadav and Banerji (2024) recommends that digital transformation implemented through training, mentoring, and digital skills development successfully improves the understanding and operational capabilities of women-owned MSMEs in using digital financial applications. This indicates that basic digital skills still need to be systematically and sustainably improved so that the benefits of digital transformation can be felt equally. Beyond the Indonesian context, global research also confirms that digital inclusion remains a major challenge for women entrepreneurs in the informal sector. Barriers such as limited access to digital infrastructure, digital illiteracy, and social factors are noted as obstacles for women in maximizing the use of digital technology in their business activities (Sowmya and Pai, 2024, 2025).

### 2.2 Financial literacy and financial inclusion

Financial literacy and financial inclusion are crucial in increasing MSMEs’ access to formal services such as credit, savings, and insurance (Ha et al., 2025; Is et al., 2025; Prayoga et al., 2025). Although financial inclusion has generally improved, the literature indicates that levels of inclusion still vary by gender and level of financial literacy. MSMEs with low levels of financial literacy tend to struggle to fully access formal financial services (Perrin and Hyland, 2023b; Peter et al., 2025; Yadav and Banerji, 2024).

Empirical studies of women-led MSMEs in local contexts show that the combination of digitalization and financial literacy impacts business decision-making, financial management, and long-term business planning. This success is highly dependent on policy support and training (Pranitasari et al., 2022, 2025; Sutopo, 2025).

### 2.3 Structural barriers in women entrepreneurship

Sociocultural factors, such as women’s domestic roles, remain a barrier to women’s involvement in the modern business world. These barriers exacerbate the gap in access to digital opportunities and financial inclusion and ultimately hinder women’s ability to fully capitalize on economic opportunities (Dzulkepli, 2021; Saluja et al., 2023; Sowmya and Pai, 2025).

From a sociological perspective, women’s involvement in the digital ecosystem is inextricably linked to the influence of domestic ideology. This ideology constructs women as primarily responsible for the domestic sphere, which in the Indonesian context manifests through cultural expectations of mothers and wives (Pranitasaria et al., 2026; Setyowati, 2020). This creates unique structural challenges for women-owned MSMEs, where digital transformation often does not reduce workloads but instead creates a “digital double burden.”

This situation triggers role conflict, namely the tension between professional demands as entrepreneurs and static domestic demands (Kailasapathy and Metz, 2012). As a result, digital technology is often domesticated to support activities aligned with household duties, ultimately limiting women’s economic mobility and agency in achieving optimal business performance (Cong et al., 2024; Haddon, 2017). This understanding broadens the sociological debate that digital inclusion for women is not simply a matter of technical access, but rather a matter of deconstructing cultural barriers that limit their access to formal economic institutions.

### 2.4 Digitalization and gendered labor regimes

A sociological perspective emphasizes that technology does not operate in a social vacuum. For women in MSMEs, the digitalization process often experiences a “domestication,” where digital tools (such as social media for business) are forced to adapt to household routines (Haddon, 2017). This creates conditions where technology does not liberate, but instead increases workloads, blurring the boundaries between business and family time. This phenomenon demonstrates that digital transformation is not simply about adopting tools, but rather about changing work regimes that often reinforce the double burden for women (Cong et al., 2024).

While various studies have examined the relationship between digital literacy, financial inclusion, and MSME performance, most studies still use quantitative approaches that focus on the relationships between individual variables. This approach often fails to explain how structural factors such as gender norms, domestic roles, and limited access to economic networks influence the lived experiences of women in MSMEs in utilizing digital technology and financial services. Therefore, a more contextual approach is needed to understand the social dynamics that shape the digitalization process and financial inclusion in women’s entrepreneurship.

### 2.5 Theoretical foundation and hypothesis development

This research is based on three main theoretical perspectives that explain the relationship between capabilities, motivation, and financial behavior in the context of women-owned MSMEs.

First, the Capability Approach proposed by Robeyns and Byskov (2025) distinguishes between capabilities and functioning’s. Capabilities refer to the real opportunities an individual has to achieve their desired life goals, while functioning’s are the actual achievements achieved. In the context of women MSMEs, the possession of resources such as digital literacy or financial access does not necessarily automatically result in optimal business performance if they cannot be converted into capabilities. This conversion process is often influenced by structural barriers such as limited access to training, social norms, and unequal economic opportunities. Therefore, digital literacy and financial support are positioned in this study as factors that strengthen women’s capabilities in managing their businesses.

Second, Self-Determination Theory (SDT) developed by Ryan and Deci (2023) explains that individual motivation is influenced by the fulfillment of three basic psychological needs: autonomy (the ability to make decisions independently), competence (the ability to carry out tasks effectively), and relatedness (connectedness to the social environment). In this study, self-development motivation reflects the intrinsic drive of female MSME owners to improve their skills and knowledge. Higher levels of digital literacy and financial knowledge are expected to strengthen their sense of competence and autonomy, thereby driving increased motivation to develop their businesses.

Third, the theory of financial behavior, as proposed by Lusardi and Mitchell (2014) emphasizes that financial knowledge plays a crucial role in shaping individuals’ financial attitudes and behaviors. In this study, financial behavior is operationally defined as the ability of MSME owners to manage business finances effectively, including budget planning, saving habits, debt management, and investment decision-making. Individuals with better financial knowledge tend to have more rational and responsible financial behaviors, which ultimately impact business sustainability.

The integration of these three theories forms a conceptual framework explaining that digital literacy and financial knowledge act as antecedent variables influencing self-development motivation and financial behavior. Specifically, digital literacy enables MSME owners to access financial information and digital financial services, thereby enhancing financial knowledge. Furthermore, digital literacy and financial knowledge also contribute to increased self-development motivation, as individuals become more confident and feel more capable in managing their businesses. Furthermore, self-development motivation plays a role in encouraging the application of knowledge into practical practice, thus influencing financial behavior. Good financial behavior then becomes a key factor in improving MSME performance, as effective financial management will support business growth and sustainability.

Based on this theory, this study develops several hypotheses explaining the relationship between individual capacity, economic practices, and women’s business performance within the context of the structural limitations they face. The hypotheses developed in this study are:

1. Digital Access and Literacy → Self-Development Motivation.
  The development of digital technology provides opportunities for women-owned MSMEs to access business information, expand market networks, and enhance entrepreneurial capacity. From a digital entrepreneurship perspective, technology functions not only as a tool but also as an enabler, encouraging opportunity exploration, innovation, and continuous learning (Nambisan, 2017). Furthermore, based on the Self-Determination Theory developed by Deci and Ryan (2000), access to resources and knowledge will increase an individual’s perceived competence, ultimately boosting intrinsic motivation.
  Empirical studies show that the adoption of digital technology significantly increases entrepreneurial motivation and engagement, especially when supported by individual capabilities and learning processes. Other studies have also found that digital literacy positively influences women’s motivation, empowerment, and capacity to develop businesses (Chiu, 2022; Cong et al., 2024; Feng et al., 2023; Plečko et al., 2023; Yu et al., 2022; Zhang, 2025). Furthermore, access to digital and economic resources strengthens individual autonomy and competence, which are key drivers of self-development motivation (Andriamahery and Qamruzzaman, 2022; Kang et al., 2023). Therefore, the higher the level of digital access and literacy, the higher the self-development motivation among women-owned MSMEs.

*H1:* Digital access and literacy have a positive effect on self-development motivation.

2. Financial Support → Self-Development Motivation.
  Financial support plays a crucial role in fostering self-development motivation, particularly among female MSME owners. The availability of financial resources, whether in the form of business capital, access to credit, or government program assistance, provides a sense of economic security, encouraging individuals to invest in capacity building, such as participating in training, developing digital skills, and expanding business knowledge. From a Self-Determination Theory perspective, adequate external support can strengthen intrinsic motivation because individuals feel they have control and the opportunity to develop (Deci and Ryan, 2000). Furthermore, financial constraints are often a major barrier for female MSME owners in accessing education and training. Therefore, when these barriers are reduced, motivation to develop significantly increases (Banerjee and Duflo, 2011; Karlan et al., 2014). Other research also shows that access to financing positively contributes to increasing individual capacity and readiness to learn and innovate in business (Demirgüç-Kunt et al., 2018). Thus, the higher the level of financial support received, the higher the individual’s motivation to pursue continuous self-development.

*H2:* Financial support has a positive effect on self-development motivation.

3. Self-Development Motivation → Digital Marketing.
  Self-development motivation drives entrepreneurs to improve business skills and adopt technological innovations in their entrepreneurial activities. Individuals with strong entrepreneurial motivation tend to be more proactive in utilizing digital technology, including social media and e-commerce platforms, to expand markets and increase business competitiveness. In the context of digital entrepreneurship, intrinsic motivation plays a crucial role in driving opportunity exploration and the adoption of technology-based innovations.
  Empirical studies show that individual motivation significantly influences entrepreneurial behavior and the adoption of digital technology in business. Research has found that entrepreneurial motivation drives the intention and use of digital technology in business activities, including digital platform-based marketing strategies (Dwivedi et al., 2021; Feng et al., 2023; Nambisan, 2017). Furthermore, motivational factors have also been shown to play a role in driving digital transformation in MSMEs, including the use of social media and e-commerce as marketing tools (Alam et al., 2022; Setyaningrum et al., 2023). Therefore, the higher the self-development motivation, the higher the tendency of MSMEs to adopt digital marketing.

*H3:* Self-development motivation has a positive effect on digital marketing.

4. Self-Development Motivation → Financial Knowledge.
  Self-development motivation drives entrepreneurs to improve their competencies and acquire new knowledge, including in business financial management. MSMEs with strong entrepreneurial motivation tend to be more active in seeking information related to financial management, capital management, and investment strategies. In the context of women’s entrepreneurship, the motivation to learn and improve their capacity can strengthen their understanding of financial concepts and enhance their business competencies.
  Empirical studies show that intrinsic motivation plays a crucial role in driving learning behavior and enhancing individual capacity. Research has found that entrepreneurial motivation significantly improves an individual’s ability to acquire knowledge and develop skills relevant to business activities (Fatima et al., 2020; Feng et al., 2023; Phung and Tran, 2023). Furthermore, motivation supported by access to resources also contributes to improving financial literacy and competency, particularly among women (Andriamahery and Qamruzzaman, 2022; Lusardi and Mitchell, 2014). Therefore, the higher the self-development motivation, the higher the level of financial knowledge among female MSMEs.

*H4:* Self-development motivation has a positive effect on financial knowledge.

5. Financial Knowledge → Financial Attitude.
  Financial knowledge is an individual’s ability to understand basic financial management concepts such as financial planning, capital management, and investment decision-making. Improved financial knowledge can foster a more positive attitude toward financial management, including savings, risk management, and long-term financial planning.
  Numerous studies have shown that increased financial literacy contributes to the development of more rational and responsible financial attitudes in managing economic resources. Individuals with higher levels of financial knowledge tend to have wiser attitudes toward financial decision-making and are better able to manage risk and plan financially effectively (Alshebami et al., 2022; Demirgüç-Kunt et al., 2018; Kurniawan et al., 2025; Lusardi and Mitchell, 2014; OECD, 2020).

*H5:* Financial knowledge has a positive effect on financial attitudes.

6. Financial Attitude → Financial Behavior.
  Financial attitude reflects an individual’s attitude toward managing financial resources, which influences their economic decision-making. A positive financial attitude can encourage individuals to implement more disciplined financial management practices, such as transaction recording, cash flow management, and business financial planning. In the context of MSMEs, sound financial attitudes play a crucial role in shaping more rational and sustainable financial behavior.
  Theoretically, the relationship between attitude and behavior is explained in the Theory of Planned Behavior, which states that individual attitudes are the primary determinant in shaping actual behavior (Ajzen, 1991). Several empirical studies also show that financial attitudes have a significant relationship with individual financial behavior in small business management. Individuals with positive financial attitudes tend to exhibit more responsible and planned financial behavior (Alshebami et al., 2022; Kurniawan et al., 2025; Lusardi and Mitchell, 2014; OECD, 2020; Xiao and O’Neill, 2016).

*H6:* Financial attitude has a positive effect on financial behavior.

7. Digital Marketing → Financial Performance.
  Utilizing digital marketing enables MSMEs to expand their market reach, increase product visibility, and strengthen relationships with consumers. Through digital platforms such as social media and marketplaces, small businesses can access a wider market with relatively low marketing costs. In the context of digital entrepreneurship, technology acts as an enabler, supporting marketing innovation and expanding business opportunities (Nambisan, 2017).
  In the context of women’s entrepreneurship, the adoption of digital technology can also help reduce structural barriers to market access and strengthen women’s economic opportunities. Studies show that the use of digital platforms contributes to increasing women’s empowerment and participation in economic activities (Saluja et al., 2023). Furthermore, various empirical studies have found that the use of digital marketing strategies, particularly through social media and online platforms, positively impacts business performance, brand visibility, and MSME business success (Cardella and Hernández-sánchez, 2020; Dwivedi et al., 2021; Setyaningrum et al., 2023).

*H7:* Digital marketing positively influences the financial performance.

8. Financial Behavior → Financial Performance.
  Good financial behavior is a crucial factor in improving business performance. Effective financial management practices, such as cash flow management, financial record keeping, and investment planning, enable MSMEs to optimize resource use and maintain financial stability. In the context of women’s entrepreneurship, good financial behavior also plays a role in strengthening business sustainability and increasing business resilience. Research shows that effective financial behavior positively contributes to improving business performance and the success of MSMEs (Andriamahery and Qamruzzaman, 2022; Cardella and Hernández-sánchez, 2020).

*H8:* Financial behavior positively influences financial performance.

**Figure 1:**
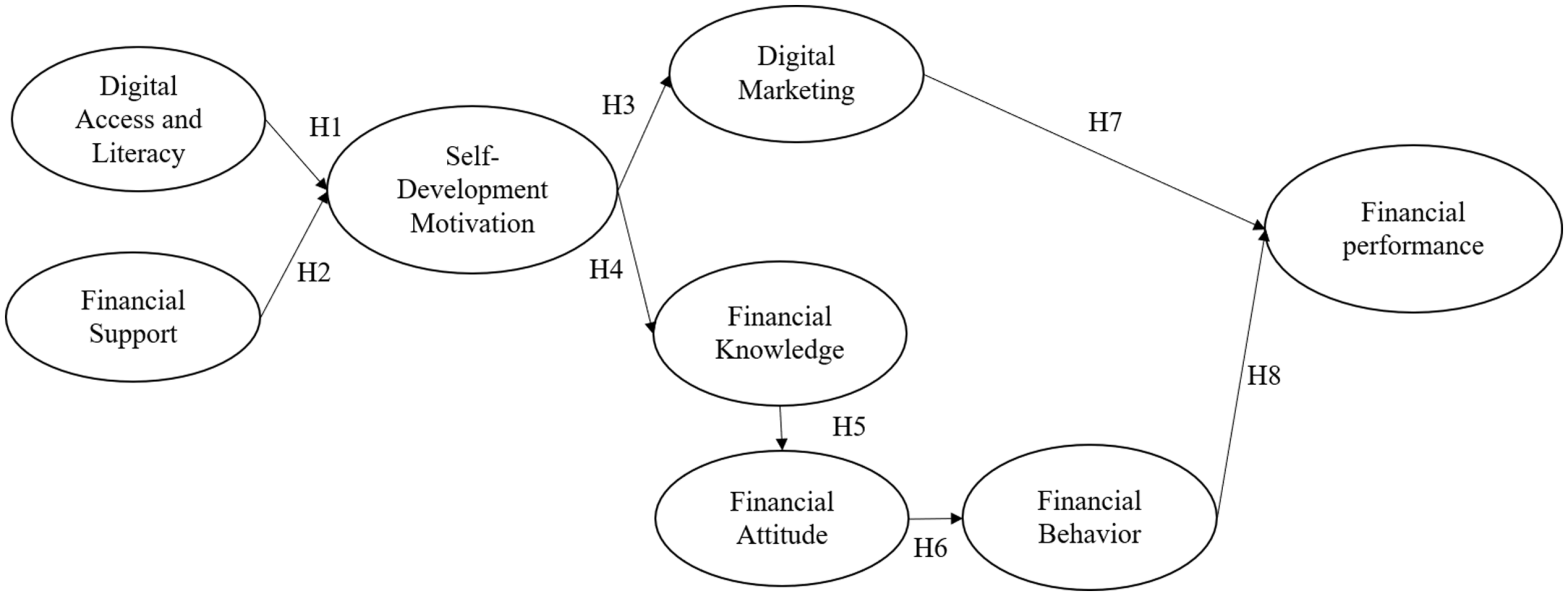
Research framework.

The variables in this study consist of independent variables, namely digital access and literacy, and financial support. Mediating variables include self-development motivation, financial knowledge, financial attitudes, financial behavior, and digital marketing. Meanwhile, the dependent variable in this study is the financial performance of women-owned MSMEs. The relationship between these variables is depicted in the conceptual model in Figure 1.

## 3 Research method

This research employed a mixed methods approach with a convergent parallel design, where quantitative and qualitative data were collected in parallel to gain a more comprehensive understanding of the phenomenon under study.

The quantitative component aimed to examine the structural relationships between digital literacy, financial literacy, financial behavior, and business performance using Structural Equation Modeling (SEM). SEM was chosen because it can analyze simultaneous relationships between multiple latent constructs while accounting for measurement error (Hair et al., 2017).

Meanwhile, the qualitative component was conducted through in-depth interviews and focus group discussions with female micro-entrepreneurs. Qualitative data analysis was conducted using thematic analysis developed by Virginia Braun and Victoria Clarke, which enabled researchers to identify patterns of social experiences and structural barriers faced by respondents.

The integration of quantitative and qualitative data was carried out at the interpretation stage of the research results. The results of the quantitative analysis were used to identify patterns of relationships between variables, while the qualitative findings provided contextual explanations of the social and institutional factors influencing these relationships (Creswell and Creswell, 2022).

The population in this study was approximately 1,500 women-owned MSMEs assisted by the Indonesian Entrepreneurship Professional Association. The sample size was determined based on the Krejcie and Morgan (1970) table, resulting in a sample size of 250 respondents. A purposive sampling approach was used to select respondents, with the following criteria: MSMEs must be led by women, have been actively operating for at least 1 year, and have participated in a mentoring program.

The research data consisted of primary and secondary data. Primary data were obtained through a structured questionnaire to measure Data Access and Literacy, Financial Knowledge, Financial Behavior, Financial Attitudes, Financial Support, Self-Development Motivation, Digital Marketing, and Financial Performance. In-depth interviews with 20 women MSME owners and Focus Group Discussions (FGDs) with relevant stakeholders were conducted to explore experiences, challenges, and institutional contexts. Secondary data were obtained from government reports, publications from international institutions, and reputable academic literature.

The research instruments were adapted from various sources validated in the literature. Digital literacy was adapted from Van Deursen and Van Dijk (2014) and Van Dijk (2020), and the DigComp framework (Judith and Romina, 2025). Financial knowledge, attitudes, and behaviors were based on the OECD (2021) and Lusardi and Mitchell (2014). Financial support was adapted from the World Bank (2020) and Brush et al. (2019). Self-development motivation was based on the Self-Determination Theory of Ryan and Deci (2017), while digital marketing was adapted from Chaffey and Ellis-Chadwick (2019). Financial performance was measured using indicators developed by Venkatraman and Ramanujam (2008) and OECD (2021). All items were measured using a 5-point Likert scale and underwent adaptation and initial validity testing. The research instrument is presented in Appendix 1.

### 3.1 Quantitative data analysis

Quantitative data were analyzed using the Partial Least Squares Structural Equation Modeling (PLS-SEM) approach with the assistance of SmartPLS version 4 software. PLS-SEM was chosen based on the consideration that this method is more suitable for complex models with many latent constructs and does not require a strict normal data distribution (Hair et al., 2017; Hair et al., 2021).

The analysis was conducted in two main stages: evaluation of the measurement model (outer model) and the structural model (inner model).

#### 3.1.1 Evaluation of the measurement model (outer model)

The evaluation of the measurement model was conducted to ensure construct validity and reliability, with the following criteria:

- Convergent validity was evaluated using Convergent Validity with a criterion of ≥ 0.70 and Average Variance Extracted (AVE) with a criterion of ≥ 0.50.
- Discriminant validity was tested using the Fornell-Larcker criterion with a HTMT value of < 0.90.
- Internal reliability was measured using Cronbach’s Alpha and Composite Reliability (CR), with a threshold value of ≥ 0.70.

#### 3.1.2 Structural model evaluation (inner model)

The structural model was analyzed to test the relationships between constructs using:

- Effect size (f^2^) to assess the contribution of each independent variable.
- Coefficient of determination (*R*^2^) to measure the model’s ability to explain variance in the dependent variable.
- Model fit was evaluated using the Standardized Root Mean Square Residual (SRMR) value, with a value of <0.08 indicating a good model (Hair et al., 2021).
- Path coefficients to determine the direction and strength of the relationships.
- t-statistics and *p*-values obtained through the bootstrapping procedure (5,000 resamplings).

### 3.2 Qualitative data analysis

Qualitative data analysis in this study was conducted using a thematic analysis approach to identify, analyze, and interpret patterns or themes emerging from in-depth interviews and focus group discussions (FGDs). All data was first transcribed verbatim to maintain the integrity of the information.

The analysis process involved several stages. First, researchers familiarized themselves with the data by repeatedly reading the interview transcripts to understand the overall context. Second, coding was conducted using a combination of deductive and inductive approaches. In the deductive stage, initial codes were developed based on the research conceptual framework, such as digital literacy, financial knowledge, financial attitudes, financial behavior, and self-development motivation. Next, inductive open coding was conducted to capture new themes that emerged directly from the field data.

To increase the reliability of the analysis, the coding process was conducted by two researchers independently. The coding results were then compared and discussed to reach inter-coder agreement. Differences in interpretation were resolved through discussion until a consensus was reached, thus minimizing subjective bias.

Data collection continued until data saturation was reached, when additional interviews no longer yielded significant new information or themes. This indicated that the data obtained was sufficient to address the research objectives.

This research also considered trustworthiness aspects to ensure the quality of qualitative data, including:

1. Credibility, conducted through data triangulation (interviews and focus group discussions) and the use of direct quotes from respondents.
2. Dependability, conducted through systematic documentation of the analysis process.
3. Confirmability, conducted through discussions between researchers to ensure data-based interpretations.
4. Transferability, conducted through detailed presentation of the research context.

Furthermore, the researchers considered reflexivity by recognizing the potential for researcher bias in the data interpretation process and striving to maintain objectivity through team discussions and traceability of the analysis process.

The results of the analysis were then organized into main themes that were used to explain and deepen the quantitative findings within a mixed methods framework, thus providing a more comprehensive understanding of the phenomena studied.

## 4 Results and discussion

### 4.1 Respondent characteristics

The majority of female MSMEs in this study were in the productive age group of 30–49 years (52.8%), with secondary to higher education levels (high school/vocational school and diploma/bachelor’s degree reaching 82.8%). Most respondents were married (72.0%), had family responsibilities (82.8%), and 43.2% served as heads of households, demonstrating the strong dual role of women as household managers and business owners. These findings align with previous research showing that the average age of female MSME owners is within the productive age range and the majority have a relatively good formal education (Tobing et al., 2024; Tobing and Shihab, 2024). This situation indicates that empowering female MSMEs requires consideration of time flexibility, ease of technology adoption, and business management efficiency.

In terms of business, women-owned MSMEs are dominated by the food and beverage sector, with 83.2% of businesses being under 10 years old and the majority still micro-scale, reflected in monthly revenues below IDR 25,000,000 (76.0%) and a limited number of employees. Business capital generally comes from personal funds, although more than half of respondents have business permits (61.2%), indicating potential for business development and access to formal financing. This reality is consistent with national data showing that micro-enterprises dominate the MSME population in Indonesia, with a much higher proportion of units than small and medium enterprises as a whole (Kadin, 2026).

Overall, these findings indicate that women-owned MSMEs are still predominantly household-based micro-enterprises, but they have potential for growth, supported by relatively high levels of education and awareness of business legality. Therefore, strengthening digitalization and financial inclusion are important strategies for improving the performance, business sustainability, and economic resilience of women-owned MSMEs. This finding is in line with research findings showing that digitalization and financial literacy drive market expansion, operational efficiency, and better business decision-making for women-owned MSMEs, thus contributing to inclusive and sustainable economic empowerment (Naurunnisa, 2025).

### 4.2 Descriptive analysis

The results of the descriptive analysis are presented in Table 1, which shows the most common respondent responses.

**Table 1:** Discriminant validity (Fornell-Larcker Criterion).

Variable	Digital access & literacy	Digital marketing	Financial attitude	Financial behavior	Financial knowledge	Financial performance	Financial support	Self-development motivation
Digital access & literacy	0.809							
Digital marketing	0.393	0.879						
Financial attitude	0.609	0.497	0.895					
Financial behavior	0.771	0.487	0.793	0.872				
Financial knowledge	0.803	0.432	0.535	0.657	0.821			
Financial performance	0.337	0.570	0.553	0.497	0.400	0.904		
Financial support	0.485	0.552	0.636	0.632	0.545	0.662	0.824	
Self-development motivation	0.502	0.476	0.696	0.647	0.601	0.758	0.617	0.848

Table 2 shows that female MSMEs generally have a fairly good understanding of the benefits of digital technology and financial services, and demonstrate positive entrepreneurial motivation and attitudes. Digital literacy and financial knowledge are at an adequate level, particularly regarding digital transactions and business development orientation. These results align with research by Kurniasari and Lestari (2024) and Bakhtiar et al. (2022) who found that female MSMEs in Indonesia have an understanding of digital technology and financial services. Social support from family and the community also plays a crucial role in sustaining business continuity, consistent with research by Arif and Hamid (2023) and Luawo (2025) which showed that family social support and community networks contribute significantly to improving business performance and self-confidence among female MSMEs. However, the use of digital technology in daily operations and marketing is not yet optimal, and financial recording and management are still inconsistent. Several previous studies have also shown that digital technology utilization in daily operations and marketing is not yet optimal. The main obstacles reported include low digital literacy and limited technical capabilities of business actors in adopting digital systems comprehensively (Ayudia et al., 2025; Mardani et al., 2026; Pranitasari et al., 2024a; Saleh et al., 2025).

**Table 2:** Distribution of respondent responses.

No	Variables	Findings
1	Digital access and literacy	Respondents understand the benefits of technology, but its use in daily operations is not yet optimal.
2	Financial knowledge	A good understanding of digital financial transactions and macroeconomic issues affecting businesses, but access to formal financial products remains limited.
3	Financial behavior	Respondents are open to professional input, but financial record-keeping is not yet consistent.
4	Financial attitudes	Respondents have a clear vision and future orientation, but they are cautious about risks.
5	Financial support	The social environment plays a significant role in supporting businesses, but access to formal capital remains a major obstacle.
6	Self-development motivation	High motivation but difficulty expanding market reach
7	Digital marketing	They believe e-commerce platforms can increase sales, but implementation of platform use is still lacking.
8	Financial performance	Increased market reach but not increased number of employees

Access to formal financing remains a major obstacle, although respondents expressed openness to financial services and digital innovation. Several studies also state that access to formal financing remains a major obstacle faced by MSMEs, due to complex administrative requirements and low managerial capacity of business actors in managing risk and meeting conventional bank criteria (Budiman et al., 2025; Lestari, 2025; Wahjono and Kristijadi, 2025). Digital marketing is perceived as capable of expanding market reach, but has not yet had a significant impact on increasing business scale and employment. These findings indicate that women-owned MSMEs have significant potential for growth if supported by empowerment strategies that integrate digitalization, financial inclusion, and sustainable managerial capacity building. These findings align with findings by Atiqah (2024), Pratama et al. (2025), and Sunggara et al. (2022) that many MSMEs still face gaps in their use of digital marketing, including low digital literacy, limited technical skills in content management, and a lack of resources to optimize these strategies.

### 4.3 Quantitative data processing

#### 4.3.1 Measurement model evaluation (outer model)

**Figure 2:**
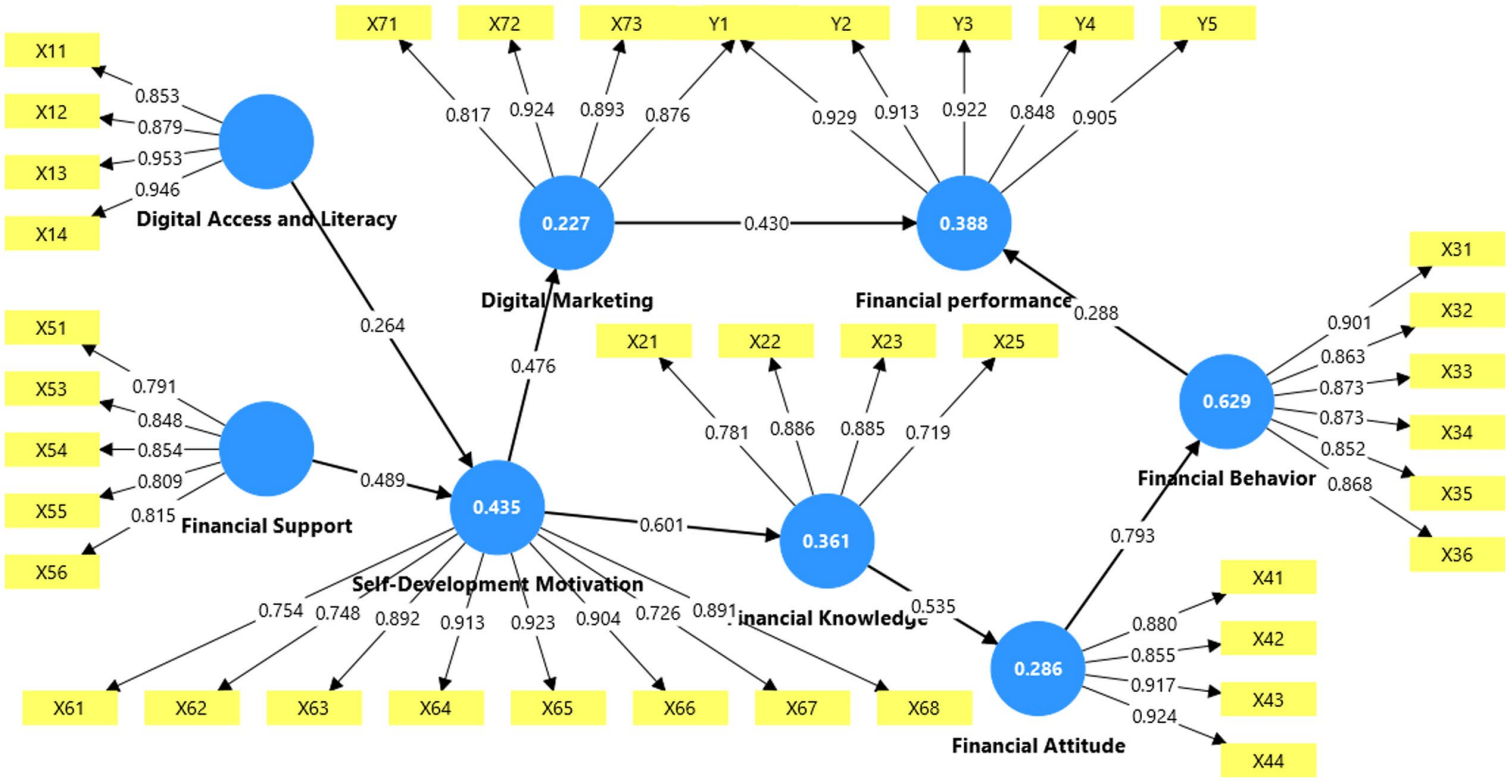
Convergent validity.

The measurement model evaluation was conducted to ensure construct validity and reliability. The convergent validity test obtained all outer loadings >0.70 after the calculation in stage 3, as presented in Figure 2.

The discriminant validity test used the Fornell-Larcker criterion with an HTMT value of <0.90. Table 1 shows that all values were <0.90.

The reliability test results showed that all research variables had Cronbach’s alpha, Composite Reliability (rho_a), and Composite Reliability (rho_c) values exceeding the threshold of 0.70. Therefore, it can be concluded that all constructs have excellent internal consistency and are reliable for use in further analysis. Furthermore, the validity test, using the Average Variance Extraced (AVE), met the criteria of >5.0. These results are presented in Table 3.

**Table 3:** Construct reliability and validity.

Variable	Cronbach’s alpha	Composite reliability (rho_a)	Composite reliability (rho_c)	AVE
Digital access and literacy	0.929	0.938	0.950	0.826
Digital marketing	0.902	0.928	0.931	0.772
Financial attitude	0.917	0.926	0.941	0.800
Financial behavior	0.937	0.940	0.950	0.760
Financial knowledge	0.836	0.853	0.891	0.674
Financial support	0.882	0.882	0.914	0.679
Financial performance	0.944	0.944	0.957	0.817
Self-development motivation	0.943	0.954	0.953	0.718

#### 4.3.2 Structural model evaluation (inner model)

The structural model was analyzed by assessing model fit using the Standardized Root Mean Square Residual (SRMR) value, with a value of <0.08 indicating a good model (Hair et al., 2021). The model in this study had an SRMR of 0.072, indicating a fairly good level of fit.

The coefficient of determination (*R*^2^) to measure the model’s ability to explain variance in the dependent variable is presented in Table 4.

**Table 4:** Coefficient of determination (R²).

Variable	R-square	R-square adjusted
Digital marketing	0.227	0.224
Financial attitude	0.286	0.283
Financial behavior	0.629	0.627
Financial knowledge	0.361	0.358
Financial performance	0.388	0.383
Self-development motivation	0.435	0.430

The *R*^2^ value indicates that the model has moderate explanatory power, with the largest contribution being financial behavior (*R*^2^ = 0.629). Self-development motivation and financial performance are in the moderate category, while the other variables are relatively lower. This indicates that the model is adequately able to explain the phenomenon under study, although there are still other factors outside the model.

The effect size (f^2^) used to assess the contribution of each independent variable is shown in Table 5. The interpretation of f-square (in PLS-SEM) refers to the extent to which an exogenous variable influences the endogenous variable.

**Table 5:** Effect size (f²).

Variable	F-square
Digital access & literacy - > Self-development motivation	0.095
Digital marketing - > Financial performance	0.230
Financial attitude - > Financial behavior	0.695
Financial behavior - > Financial performance	0.103
Financial knowledge - > Financial attitude	0.401
Financial support - > Self-development motivation	0.324
Self-development motivation - > Digital marketing	0.294
Self-development motivation - > Financial knowledge	0.564

The results of the effect size (f^2^) analysis indicate that the model is dominated by internal pathways that shape individual capacity, specifically through the chain of financial knowledge → financial attitude → financial behavior. In this case, financial attitude emerges as a key link that strongly translates knowledge into actual behavior.

Furthermore, self-development motivation plays a key role in driving capacity building, particularly in strengthening financial knowledge and adopting digital marketing. This confirms that motivational factors are a crucial foundation in the process of developing business actors’ competencies.

Conversely, external factors such as digital access and financial behavior show a relatively limited contribution to performance and are therefore not the primary determinants in the model. Overall, these findings indicate that strengthening internal aspects, particularly motivation, knowledge, and attitude, is more crucial than access factors alone.

**Figure 3:**
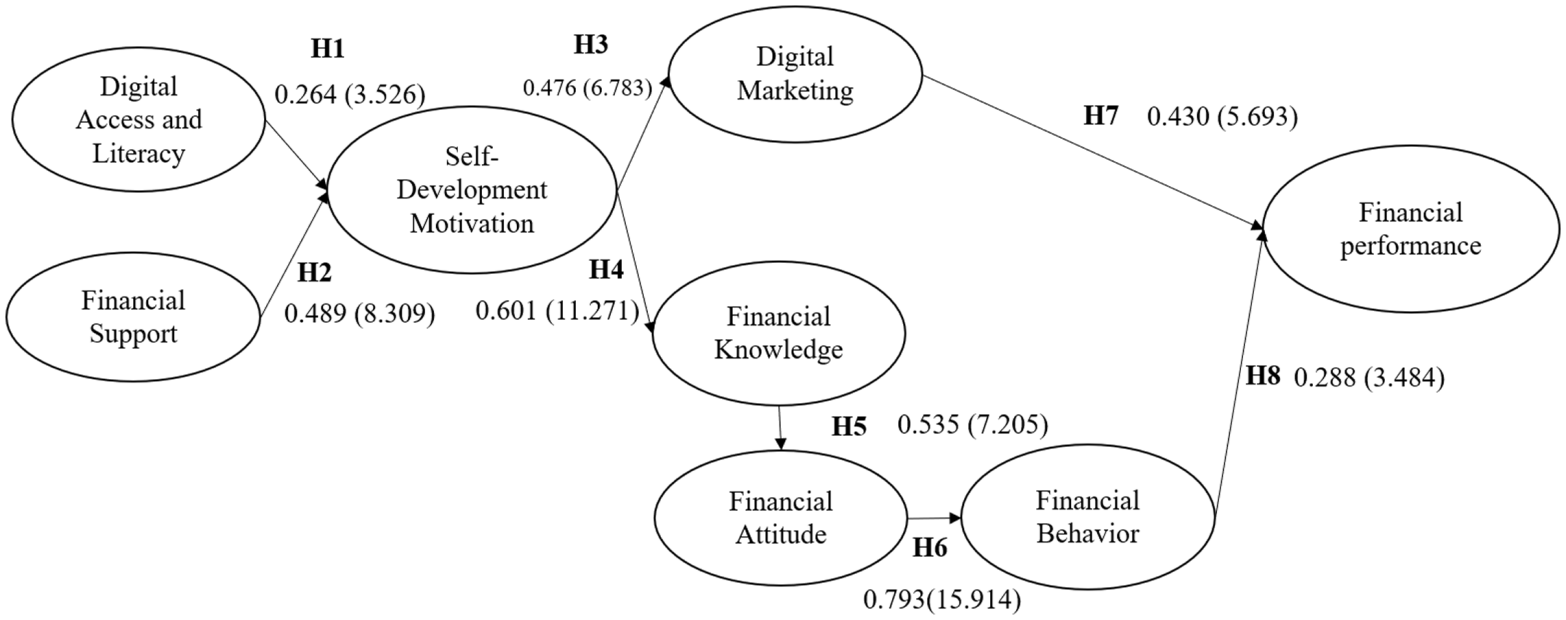
Path coefficient values and significance test (*T*-statistic).

The results of the significance test and coefficients for each pathway are depicted in Figure 3.

Figure 3 shows that the test results indicate that all relationships between variables are statistically significant (t-statistic >1.96). However, in addition to significance, the analysis also shows variation in the strength of the influence between variables. Financial support has a stronger influence on self-development motivation than digital literacy, indicating that financial factors play a dominant role in fostering an individual’s readiness to develop.

Furthermore, the path from financial knowledge to financial attitude shows the largest coefficient in the model, indicating that increased financial knowledge is a key determinant in developing positive financial attitudes.

Digital access and literacy significantly increase self-development motivation. This means that the better the respondents’ digital skills and access, the greater their drive to develop their capacity, Salim and Lubis (2025), Supartha and Desak (2017), Vaszkun and SZakács (2025), and Zhang (2025) also produced consistent research results. Financial support has a stronger influence than digital literacy on self-development motivation. This aligns with the research findings of Fatima et al. (2020), Kang et al. (2024), and Sitriani (2025), indicating that the availability of capital or financial assistance is a crucial factor in encouraging individual development. Self-development motivation is simultaneously influenced by digital and financial factors, with the latter playing a more dominant role.

Self-development motivation drives individuals to adopt and optimize digital marketing, Plečko et al. (2023) and Ridwan and Zaki (2023) also obtained similar research results. Likewise, high motivation tends to actively improve financial knowledge (Phung and Tran, 2023). Self-development motivation acts as a key mediating variable in increasing digital and financial capacity.

Good financial knowledge shapes positive financial attitudes. This is the strongest influence in the model, indicating that financial attitudes play a significant role in determining actual financial behavior. Financial knowledge influences financial attitudes and, in turn, financial behavior, forming a complete chain of financial literacy (Dityarukmana, 2025; Hidayat et al., 2023).

The implementation of digital marketing has been shown to improve financial performance, as research has shown (Jung and Shegai, 2023; Liang, 2025). Healthy financial behavior also contributes positively to financial performance (Dityarukmana, 2025; Hidayat et al., 2023), although the effect is smaller than that of digital marketing.

The research results show that self-development motivation plays an important role as a mediator in connecting digital access and financial support with increased digital capabilities and financial literacy, which ultimately have a positive impact on financial performance.

### 4.4 Qualitative data

Qualitative data was obtained through in-depth interviews and FGDs to deepen understanding of the quantitative results and identify the social and institutional contexts that influence the performance of women’s MSMEs. Thematic analysis showed that the main obstacles for women’s MSMEs lie not in the absence of programs, but rather in limited access, capacity, and the mismatch between policies and the actual conditions of business actors.

#### 4.4.1 Access to digital and financial literacy

Key questions asked of respondents:

1. What is your experience using digital technology for marketing and business financial recording?
2. Have you ever participated in training related to digital marketing or business financial management?

Summary of respondents’ answers:

Most respondents stated that they lack basic skills in digital marketing and digital financial recording. Their use of technology is generally limited to simple communication through instant messaging applications, without the use of digital marketing platforms or financial applications. Respondents stated that limited knowledge and a lack of confidence are the main reasons for not optimally utilizing technology, even though digital infrastructure is available.

This research further explores the capacity-performance gap, a condition where increased digital and financial literacy does not automatically improve the economic performance of women’s MSMEs. Through Pierre Bourdieu’s lens, this phenomenon occurs due to inequality in the conversion of social capital into economic capital. Qualitative findings indicate that strategic information regarding government policies and market access tends to be concentrated in exclusive networks (bonding social capital), such as communities with close ties to village officials or local elites.

Consequently, female MSMEs outside these circles experience social exclusion in the distribution of information. This demonstrates that business performance is hampered not by low individual capacity, but by the individual’s habitus and position within an unequal social structure (Pratono, 2018; Setyowati, 2020). The theoretical advancement offered by this research is that financial inclusion will not achieve maximum performance as long as the distribution of social capital remains discriminatory and exclusive at the grassroots level (Pratono et al., 2020).

#### 4.4.2 Access to formal financing

Key questions asked to respondents:

1. Have you ever applied for credit from a formal financial institution? Why yes or no?
2. What are the main obstacles you face in accessing bank financing?

Summary of Respondents’ Responses:

The majority of respondents had never accessed bank credit. The main obstacles identified included the lack of collateral in their own name, incomplete business legality, and a lack of understanding of formal financial products. Furthermore, there was a perception that bank credit was complicated and risky, even contrary to religious values, leading respondents to prefer using personal capital or family support.

#### 4.4.3 Access to empowerment programs

Key questions asked of respondents:

1. Are you aware of or have you participated in MSME empowerment programs from the government or other institutions?
2. How did you obtain information regarding business training or mentoring?

Summary of Respondents’ Responses:

It can be concluded that there is a gap in access to information regarding empowerment programs. Respondents who are members of MSME communities or associations have relatively easy access to information and mentoring. Conversely, unorganized women entrepreneurs experience limited access to information and are unaware of the existence of digital training programs or subsidized financing. This indicates that program distribution still relies on social networks and institutions.

#### 4.4.4 Dual roles and limited participation

Key questions asked of respondents:

1. What are your main obstacles in participating in business training or mentoring?
2. How do you divide your time between your business and household responsibilities?

Summary of respondents’ answers:

The majority of respondents emphasized that their dual roles as both business owner and household manager limit their participation in training and mentoring. Inflexible schedules and difficult-to-reach locations are major obstacles, although respondents expressed a strong interest in increasing their business capacity.

The results of the FGD conducted with local government, banking, and MSME communities revealed shared perceptions and experiences that reinforced the findings of the in-depth interviews. In the focus group discussions, participants agreed that various programs for empowering women’s MSMEs are available, but their benefits have not yet been fully felt by business owners.

The FGD participants assessed that the main problem is not the lack of programs, but rather the information distribution mechanisms, inflexible program designs, and the lack of ongoing mentoring. Several participants revealed that information about training and financing is more often received by MSME owners who are already members of the community or have close ties with local officials, while women entrepreneurs outside these networks tend to receive less information.

In the discussions, participants also shared the view that digital and financial literacy training is often overly technical, short-term, and not tailored to the participants’ initial skill level. As a result, the training has not been able to increase participants’ confidence and courage to practice digital technology or access formal financial institutions.

The FGD results also confirmed that women’s dual roles are a structural issue affecting participation rates. Participants collectively proposed that future empowerment programs be implemented with flexible schedules, in locations close to their homes, or through community-based mentoring, to better align with the domestic responsibilities inherent to female MSMEs.

Overall, the results of the interviews and FGDs indicated that the challenges facing female MSMEs are multidimensional, encompassing limited individual capacity (literacy and self-confidence), structural barriers (asset ownership and gender roles), and institutional weaknesses (access to information and program design). These findings emphasize that empowerment efforts for female MSMEs need to go beyond a purely program-based approach and focus on strategies that are more inclusive, community-based, gender-sensitive, and adaptive to the social and economic conditions of female entrepreneurs.

### 4.5 Integration of quantitative and qualitative results

The quantitative analysis results show that financial attitude has the strongest influence on financial behavior, followed by self-development motivation in improving financial knowledge and digital marketing. These findings were further clarified through qualitative interviews.

Qualitatively, respondents emphasized that attitude toward financial management is a key factor in determining how they run their businesses. Several female MSME owners stated that awareness of separating business and personal finances and long-term planning stems not only from knowledge but also from a mindset formed through experience.

“Previously, I didn’t separate business and personal money, but after realizing the importance of managing my finances, my business became more controlled and grew” (Respondent 3).

This finding reinforces the quantitative results that financial attitude and financial behavior are the most dominant relationship in the model. Furthermore, self-development motivation also emerged as a significant factor driving individual capacity building, particularly in understanding finance and utilizing digital technology.

“I self-taught myself from the internet and participated in training so I could sell online and manage my finances better” (Respondent 7).

This aligns with the quantitative results, which show that self-development motivation has a strong influence on financial knowledge and digital marketing. However, the qualitative results also revealed that digital access alone is insufficient without a willingness to learn and adapt.

“There are already many applications, but if you’re not willing to learn, you still can’t develop” (Respondent 2).

This finding explains why in the quantitative model, the effect of digital access on motivation is relatively small, as internal factors such as motivation are more important than mere access. On the other hand, although financial behavior influences financial performance, several respondents revealed that improved business performance is also influenced by external factors such as market conditions and competition.

“Even though you manage your finances well, sometimes sales still decline due to market conditions” (Respondent 5).

This indicates that the relationship between financial behavior and performance is not entirely strong, as seen in the quantitative results with a relatively small effect size. This relationship confirms that improved financial performance does not occur immediately, but rather through a gradual process involving motivational, capability, and behavioral factors. This aligns with research conducted by Dwijayanty (2025) and Supramono et al. (2025).

Qualitative findings from in-depth interviews and FGDs provide a contextual explanation of the mechanisms of this relationship. The low levels of digital and financial literacy expressed by respondents explain why self-development motivation serves as a key mediating variable in the quantitative model. Despite the availability of digital infrastructure and programs, limited capacity and confidence prevent women’s MSMEs from optimally capitalizing on opportunities without strong internal motivation. Arifin et al. (2023), Harini et al. (2023), Juita et al. (2026), and Pranitasari et al. (2024b) concluded that women tend to lag behind in digital skills and technology participation, making psychological and behavioral factors key barriers to capitalizing on available digital opportunities.

Furthermore, qualitatively identified structural barriers to access to formal financing, such as limited asset ownership, business legality, and negative perceptions of financial institutions, reinforce the quantitative findings regarding the strong influence of financial support on self-development motivation. Financial support not only serves as a source of capital but also as a psychological factor that increases women entrepreneurs’ sense of security and readiness to develop their businesses.

The integration of both types of data also demonstrates a consistent pathway for financial knowledge, financial attitudes, and financial behavior. The statistically significant causal relationship aligns with the qualitative findings, which indicate that training and mentoring can improve financial management discipline. However, women’s dual roles and limited program flexibility are inhibiting factors in sustaining changes in financial behavior.

Overall, the integration of quantitative and qualitative findings confirms that the challenges faced by women’s MSMEs are multidimensional, encompassing individual capacity, structural barriers, and institutional barriers. These findings suggest that strategies for empowering women’s MSMEs need to be designed in an integrated, community-based, and gender-sensitive manner to optimize the impact of digitalization and financial inclusion on sustainable financial performance.

## 5 Discussion

The results of this study provide a more comprehensive understanding of the mechanisms by which digitalization and financial inclusion influence the financial performance of women-owned MSMEs. Quantitative findings indicate that digital access, literacy, and financial support do not directly impact financial performance, but rather operate through a mediating pathway involving self-development motivation, digital marketing, and a series of financial literacy strategies consisting of financial knowledge, attitudes, and behavior. This pattern confirms that improving the financial performance of women-owned MSMEs is a gradual process that requires strengthening the internal capacity of business actors, not simply the provision of infrastructure or programs, as also emphasized in the literature on capability building in MSMEs (Azcona et al., 2023; Pranitasari et al., 2024b; Saputra et al., 2024).

The role of self-development motivation as a primary mediator is a key finding of this study. Theoretically, this finding reinforces the capability approach, which states that access to resources will only be effective if individuals have the capacity and internal drive to utilize them (Robeyns and Byskov, 2025). However, this study further explores the capacity-performance gap, where increased digital literacy does not automatically correlate with economic performance. Through Pierre Bourdieu’s lens, this phenomenon occurs due to the imbalance in the conversion of social capital into economic capital. Qualitative findings indicate that strategic information regarding market access and financial assistance tends to circulate only within exclusive social networks (bonding social capital). As a result, female MSMEs outside these networks experience social exclusion, hindering their individual capacity due to unequal access based on social structures (Pratono, 2018; Setyowati, 2020). Previous research also shows that intrinsic motivation plays a significant role in technology and innovation adoption in MSMEs, particularly among female entrepreneurs (Saleh et al., 2025; Soares and Ahmad, 2025; Utomo and Setiyono, 2024; Yuwono et al., 2024; Williams and Shahid, 2016; Santos and Liguori, 2020). The qualitative findings in this study strengthen this argument by showing that limited self-confidence and feelings of inadequacy are major barriers to the use of digital technology, even though infrastructure and programs are available.

These findings indicate that the success of women-owned MSMEs is not solely determined by internal business factors but is also heavily influenced by role conflict. Sociologically, dual roles are not a technical issue of individual time management, but rather a manifestation of social norms in Indonesia that place domestic responsibilities as women’s primary responsibility (Setyowati, 2020). This explains why, despite increasing digital literacy, business performance is often held back at the micro level; women’s productive energy and time are fragmented by static, non-delegable domestic demands (Kailasapathy and Metz, 2012).

The strong relationship between financial knowledge, financial attitudes, and financial behavior confirms the literature that views financial literacy as a hierarchical process. Financial knowledge shapes more rational and long-term-oriented financial attitudes, which are subsequently reflected in more disciplined financial behavior (Dityarukmana, 2025; Hidayat et al., 2023). These findings are consistent with empirical studies on MSMEs in developing countries, which show that financial literacy indirectly impacts business performance through changes in financial attitudes and behavior (Dwijayanty, 2025; Sajuyigbe et al., 2024; Tarus and Tarus, 2024). However, the qualitative results of this study indicate that women’s dual roles and time constraints are factors that hinder the sustainability of healthy financial behavior, as also reported in research related to gender and entrepreneurship (De Clercq and Kaciak, 2021; Oridi et al., 2025).

The stronger effect of digital marketing on financial performance compared to financial behavior suggests that market expansion through digital channels is a relatively rapid path to increasing income for women-owned MSMEs. These findings align with research indicating that the adoption of e-commerce and digital marketing significantly contributes to increased sales, cost efficiency, and market access for MSMEs (Atiqah, 2024; Jung and Shegai, 2023; Liang, 2025; Sunggara et al., 2022). Sociologically, the use of digital marketing also reflects the transformation of labor regimes in the informal sector. The use of digital platforms creates a phenomenon of digital domestication, where technology does not liberate women but instead forces them to be always-on. This blurs the boundaries between work and domestic space, ultimately increasing the emotional and physical workload for female MSME owners (Cong et al., 2024). Furthermore, this study positions digital transformation in the informal economy not simply as an expansion of market reach, but as a shift in complex labor regimes. Qualitative findings indicate that, rather than providing full emancipation, digitalization often creates a new double burden. Sociologically, a phenomenon of “digital domestication” has emerged, where women are required to manage marketing content, respond to customers in real time, and monitor orders via mobile phones while simultaneously carrying out ongoing domestic work (Cong et al., 2024; Haddon, 2017).

This condition creates blurred boundaries between private and professional workspaces. As a result, this digital transformation risks reinforcing self-exploitation under the guise of work flexibility, with women shouldering a double burden that transforms into a triple burden (domestic, production, and digital management) without any deconstruction of the gender-based division of labor at the household level (Setyowati, 2020).

However, qualitative results indicate that the use of digital marketing remains partial and has not been strategically integrated, as also found in studies of women-led MSMEs (Azcona et al., 2023; Bhagat et al., 2021; OECD, 2023).

The findings regarding the strong influence of financial support on self-development motivation confirm that access to financing serves not only as a source of capital but also as a psychological factor that increases a sense of security and the courage to take business risks. Previous research has shown that limited access to formal financing for women’s MSMEs is often caused by structural barriers, such as asset ownership, business legality, and gender bias in the financial system (Allison et al., 2023; OECD, 2025; Perrin and Hyland, 2023a). Furthermore, this study extends the sociological debate on gender-based access to economic institutions by demonstrating that the formal financial system in Indonesia remains gender-blind. Collateral requirements set by financial institutions often assume equal asset ownership, even though in patriarchal household structures, productive assets are generally controlled by men (Setyowati, 2020). This creates structural barriers that prevent women from actualizing their economic agency due to dependence on their husbands’ financial authority (Rinaldo, 2018). This situation explains why negative perceptions of banking remain high, as the system ignores the sociological realities of asset ownership at the grassroots level. The negative perceptions of formal financial institutions revealed in the qualitative findings of this study are also consistent with the literature highlighting the low trust of women-owned MSMEs in formal financial institutions (Dzulkepli, 2021; Finance, 2021; Irene et al., 2025; Koomson et al., 2023).

Overall, this discussion confirms that empowering women-owned MSMEs requires an integrated and gender-sensitive approach. Digitalization and financial inclusion will only have an optimal impact if accompanied by strengthened motivation, increased financial literacy capacity, and program design that adapts to women’s social contexts. The main contribution of this study lies in providing a mixed-methods-based empirical model that not only examines the relationships between variables but also explains the mechanisms and underlying social contexts, thereby enriching the literature on women-owned MSMEs in developing countries.

## 6 Theoretical contribution

This research contributes to the socio-economic and women’s entrepreneurship literature by demonstrating that improving individual capacities, such as digital literacy and financial knowledge, does not always directly translate into improved business performance. These findings identify a capacity-performance gap, namely the gap between improved individual capabilities and the economic outcomes achieved in women’s entrepreneurial practices (Demirgüç-Kunt et al., 2025; Kabeer, 2021; Williams and Shahid, 2016).

This research expands the debate on gender-based economic inclusion by demonstrating that women’s entrepreneurial success is not solely determined by individual factors but is also influenced by structural conditions such as access to economic resources, social networks, and institutional support. In many informal economic contexts, women still face various structural barriers such as limited access to finance, asset ownership, and limited business networks, which ultimately impact their chances of business success (Demirgüç-Kunt et al., 2022; Visvizi et al., 2026).

This research contributes to the literature on women’s entrepreneurship within structural and institutional contexts by emphasizing the importance of the social and cultural environment in shaping entrepreneurial opportunities. Previous research has shown that cultural factors, family support, and social networks play a significant role in shaping the success of women’s businesses. Therefore, women’s entrepreneurship needs to be understood not only as an individual phenomenon but also as a social process influenced by economic and institutional ecosystems (Kurniawan et al., 2025).

This research adds to the literature on digital transformation in the informal economy by demonstrating that digitalization does not necessarily automatically result in economic empowerment. While digital technology opens access to market information and business opportunities, the economic benefits of digitalization depend heavily on individuals’ ability to convert digital access into economic resources and the support of broader social networks and economic institutions. Thus, digitalization needs to be understood as a social process that interacts with gender structures, cultural norms, and access to economic resources (Hunt and Samman, 2019; Safa et al., 2025).

The findings of this study indicate that women’s business success is influenced not only by individual capacities such as digital literacy and financial knowledge, but also by socioeconomic conditions and access to resources. This is in line with previous research which shows that socio-economic factors and institutional support play an important role in determining the success of female entrepreneurship (Valque et al., 2025).

## 7 Conclusion

This study aims to analyze the influence of digitalization and financial inclusion on the performance of women-owned MSMEs by considering mediating mechanisms and structural barriers. The results indicate that digitalization and financial support do not directly improve business performance, but rather through mediating variables such as self-development motivation, digital marketing, and financial behavior. In general, the results of hypothesis testing indicate that most of the relationships between variables are empirically supported, particularly the mediating pathway that strengthens business performance.

These findings confirm the existence of a capacity-performance gap, where increased individual capacity is not fully translated into economic outcomes due to structural limitations such as financial access, asset ownership, and social norms. Thus, this study makes an important contribution in explaining that the success of women-owned MSMEs is determined not only by individual capabilities but also by surrounding structural factors.

The practical implications of this study suggest that women-owned MSME empowerment programs need to integrate digital training, financial literacy, and structural support such as access to financing and gender-sensitive policies. Meanwhile, theoretically, this study strengthens the sociological approach to entrepreneurship by emphasizing the importance of the interaction between individual capacity and the structural context in determining business performance.

This study’s limitations lie in the limited sample size of assisted MSMEs, which limits the generalizability of the findings. Therefore, further research is recommended to expand the scope, employ a longitudinal approach, and more explicitly examine the role of structural variables in a quantitative model to gain a more comprehensive understanding.

### 7.1 Recommendations

Women’s MSME empowerment programs need to be designed sustainably by integrating digital training, financial literacy, and self-development motivation, not just one-off training. This is crucial given that research shows a capacity-performance gap, where increased capacity does not automatically impact business performance. Therefore, training programs should be designed flexibly and community-based to be more accessible to women who play dual roles as household managers and business owners.

The government, MSME support agencies, and financial institutions need to provide ongoing practical support to help women entrepreneurs utilize digital technology effectively. Furthermore, access to digital platforms and formal financing needs to be facilitated by simplifying requirements and providing support in administrative processes and business management.

Digital training also needs to be complemented with more applicable materials, such as digital marketing strategies, content management, and market network development. Thus, the use of digital technology not only increases individual capacity, but can also have a real impact on increasing income and expanding the market for women’s MSMEs.

## Data Availability

The raw data supporting the conclusions of this article will be made available by the authors, without undue reservation.
