# Supplementary material for: Digitalization and financial inclusion among women-led micro and small enterprises in Indonesia: an empirical perspective
Source: Front Sociol. 2026 May 14;11:1813070. doi: 10.3389/fsoc.2026.1813070 (PMC13215917; doi:10.3389/fsoc.2026.1813070)
Supplement: Supplementary file 1 [file Supplementary_file_1.pdf]

Appendix 1. Distribution of Respondents' Answers

Variables	Instruments
Digital Access and Literacy	I sell goods and services using an e-commerce platform.
	I believe using an e-commerce platform can increase my sales.
	I believe using an e-commerce platform can save costs.
	I know how to operate mobile applications.
Financial Knowledge	I can access savings and loan products offered by commercial banks and BPR.
	I know how to conduct financial transactions, whether through bank tellers, online banking, mobile banking, or QRIS.
	I know how to conduct financial transactions through financial applications.
	Compared to commercial banks and BPR, online loan interest rates are generally higher.
	I understand the impact of inflation on my business.
Financial Behavior	I maintain a clear separation of business and personal finances.
	I record/bookkeep my business transactions.
	I know my business profit periodically.
	I always look for cheaper sources of raw materials of the same quality.
	For me, low interest is more important than the speed of credit disbursement.
	I always consult/discuss with parties I consider experts to develop my business.
Financial Attitudes	I have a long-term plan for growing my business.
	I am confident that my business will be successful and sustainable in the future.
	In addition to daily living expenses, I set aside a portion of my profits for business development.
	In addition to daily living expenses, I also set aside a portion of my profits for emergencies.
	I am willing to take high risks to achieve a high level of profit.
	I can easily obtain capital loans from financial institutions.

**Variables**	**Instruments**
Financial Support	My family supports my business activities.
	I receive support and mentoring from relevant government agencies to develop my business.
	I am part of a women's/MSME community that supports business development.
	I receive equal treatment as male entrepreneurs.
	I feel that existing government policies address the special needs of women entrepreneurs.
Self-Development Motivation	I run this business with enthusiasm and sincerity.
	I am able to balance my time between business and household chores.
	I have the skills to manage my business well.
	I am capable and active in generating new ideas to attract customers.
	I have a good command of the technical skills needed to run my business.
	I am always learning new technologies that can support my business.
	I am able to reach customers from outside the region.
	I am able to analyze potential market demand for the products/services I offer.
Digital Marketing	I promote The products/services I sell through social media
	I sell goods and services using an e-commerce platform
	I believe using an e-commerce platform can increase my sales
	I believe using an e-commerce platform can save costs
Financial performance	My business's sales volume and value have increased
	My business's profits have increased
	My business's market reach has increased
	My business's number of employees has increased
	My business has adequate cash reserves
